# Preoperative clinical and tumor genomic features associated with pathologic lymph node metastasis in clinical stage I and II lung adenocarcinoma

**DOI:** 10.1038/s41698-021-00210-2

**Published:** 2021-07-21

**Authors:** Raul Caso, James G. Connolly, Jian Zhou, Kay See Tan, James J. Choi, Gregory D. Jones, Brooke Mastrogiacomo, Francisco Sanchez-Vega, Bastien Nguyen, Gaetano Rocco, Daniela Molena, Smita Sihag, Prasad S. Adusumilli, Matthew J. Bott, David R. Jones

**Affiliations:** 1 Thoracic Service, Department of Surgery, Memorial Sloan Kettering Cancer Center, New York, NY USA; 2 Thoracic Department, Peking University People’s Hospital, Beijing, China; 3 Department of Epidemiology and Biostatistics, Memorial Sloan Kettering Cancer Center, New York, NY USA; 4 Druckenmiller Center for Lung Cancer Research, Memorial Sloan Kettering Cancer Center, New York, NY USA; 5 Center for Molecular Oncology, Memorial Sloan Kettering Cancer Center, New York, NY USA

**Keywords:** Surgical oncology, Non-small-cell lung cancer

## Abstract

While next-generation sequencing (NGS) is used to guide therapy in patients with metastatic lung adenocarcinoma (LUAD), use of NGS to determine pathologic LN metastasis prior to surgery has not been assessed. To bridge this knowledge gap, we performed NGS using MSK-IMPACT in 426 treatment-naive patients with clinical N2-negative LUAD. A multivariable logistic regression model that considered preoperative clinical and genomic variables was constructed. Most patients had cN0 disease (85%) with pN0, pN1, and pN2 rates of 80%, 11%, and 9%, respectively. Genes altered at higher rates in pN-positive than in pN-negative tumors were *STK11* (*p* = 0.024), *SMARCA4* (*p* = 0.006), and *SMAD4* (*p* = 0.011). Fraction of genome altered (*p* = 0.037), copy number amplifications (*p* = 0.001), and whole-genome doubling (*p* = 0.028) were higher in pN-positive tumors. Multivariable analysis revealed solid tumor morphology, tumor SUVmax, clinical stage, *SMARCA4* and *SMAD4* alterations were independently associated with pathologic LN metastasis. Incorporation of clinical and tumor genomic features can identify patients at risk of pathologic LN metastasis; this may guide therapy decisions before surgical resection.

## Introduction

Lung adenocarcinoma (LUAD) is the most common histologic subtype of non-small cell lung cancer (NSCLC)^1^ and is associated with a higher risk of occult lymph node (LN) metastasis than other NSCLC tumors^2,3^. Combined positron emission tomography/computed tomography (PET/CT) imaging is the standard noninvasive study for LN staging^4–6^; however, it is associated with a high false-negative rate^7^. In patients with no uptake of fluorodeoxyglucose on PET/CT, the incidence of occult mediastinal LN metastases is 7–18%^8–11^. In addition, PET/CT may have reduced accuracy in patients with nodes <1 cm in short-axis diameter^12^, and false-positives can occur secondary to inflammation or infection^13^.

Previous studies have identified clinicopathologic variables associated with occult LN metastasis in patients with clinical N2-negative disease. These include centrally located tumors, large tumor size, high primary tumor maximum standardized uptake value (SUVmax), positive N1 nodes on PET/CT, and micropapillary histologic pattern^8,9,14,15^. In clinical practice, broad-panel next-generation sequencing (NGS) has increasingly been used to elucidate tumor biology, identify targetable driver-gene perturbations, and inform prognoses for patients with NSCLC^16^. To date, no study has used NGS to determine the risk of pathologic LN metastasis in patients with LUAD. Importantly, it is unknown whether this information could potentially guide the order of first line therapy (i.e., systemic vs. local) in the future. To bridge this knowledge gap, we examined tumor genomic factors in patients with clinically N2-negative (cN0-1) LUAD and assessed their association with pathologic LN metastasis identified at surgery.

## Results

### Clinicopathologic characteristics

In total, 426 patients met the inclusion criteria; 60% (*n* = 255) were men, and 74% (*n* = 316) were current or former smokers. Clinicopathologic features are summarized in Table 1. A majority of patients had clinical stage I disease (*n* = 334 [78%]); 22% (*n* = 92) had clinical stage II disease. On pathologic review, 80% of resected tumors (*n* = 341) were pN0 (pN-negative), and 20% (*n* = 85) were pN1 or pN2 (pN-positive). On diagnostic CT, 59% (201/341) of pN-negative tumors and 92% (78/85) of pN-positive tumors had solid morphologic appearance (*p* < 0.001). Median primary tumor SUVmax was 3.5 (IQR, 1.8–6.5) for pN-negative tumors and 7.8 (IQR, 4.6–10.5) for pN-positive tumors (*p* < 0.001). Invasive staging was performed in 10% of patients with clinical early-stage disease (*n* = 42)—9% underwent EBUS (*n* = 38), 0.7% underwent mediastinoscopy (*n* = 3), and 0.2% underwent both (*n* = 1).

**Table 1:** Clinicopathologic characteristics.

Characteristic	pN-negative (*N* = 341)	pN-positive (*N* = 85)	*p*
Age at resection, years	69 (64–74)	69 (62–74)	0.8
Sex			
Male	212 (62)	43 (51)	0.063
Female	129 (38)	42 (49)	
Smoking status			
Never	91 (27)	19 (22)	0.5
Ever	250 (73)	66 (78)	
Pack-years (*N* = 425)	16 (0–40)	20 (3.8–45)	0.2
Tumor morphologic appearance on CT (*N* = 423)			
Nonsolid	137 (41)	7 (8)	<0.001
Solid	201 (59)	78 (92)	
Tumor location			
RUL	110 (32)	26 (31)	0.095
RML	23 (7)	2 (2)	
RLL	64 (19)	14 (16)	
LUL	84 (25)	23 (27)	
LLL	51 (15)	12 (14)	
Multiple lobes	9 (3)	8 (9)	
Tumor size on CT, cm (*N* = 425)	2.2 (1.5–3)	2.9 (1.9–3.8)	<0.001
Tumor SUVmax (*N* = 405)	3.5 (1.8–6.5)	7.8 (4.6–10.5)	<0.001
cN stage (CT and PET criteria)			
cN0	308 (90)	54 (64)	<0.001
cN1	33 (10)	31 (36)	
Clinical stage			
I	288 (84)	46 (54)	<0.001
II	53 (16)	39 (46)	
Mediastinal staging			
EBUS	16 (5)	22 (26)	<0.001
Mediastinoscopy	2 (0.6)	1 (1)	
EBUS and mediastinoscopy	0	1 (1)	
None	323 (95)	61 (72)	
Preoperative biopsy contains MIP or SOL subtype (*N* = 227)			
No	142 (82)	41 (77)	0.493
Yes	32 (18)	12 (23)	
pN status			
pN0	341 (100)	0	<0.001
pN1	0	48 (56)	
pN2	0	37 (44)	
Pathologic stage			
I	284 (83)	0	<0.001
II	49 (14)	42 (49)	
III	8 (2)	43 (51)	

Data are presented as no. (%) or median (interquartile range).

*CT* computed tomography, *EBUS* endobronchial ultrasound, *LLL* left lower lobe, *LUL* left upper lobe, *MIP* micropapillary, *PET* positron emission tomography, *RLL* right lower lobe, *RML* right middle lobe, *RUL* right upper lobe, *SOL* solid.

A median of 3 N1 stations (interquartile range [IQR], 2–3), 3 N2 stations (IQR, 2–3), and 5 total stations (IQR, 4–6) were sampled intraoperatively (Supplementary Table 1). Pathologic stages were as follows: 67% stage I (*n* = 284), 21% stage II (*n* = 91), and 12% stage III (*n* = 51). A total of 362 patients (85%) had cN0 disease, 15% of whom (54/362) had occult pathologic LN metastasis. In addition, 64 patients (15%) had cN1 disease, 48% of whom (31/64) had confirmed pathologic LN involvement. Occult pN2 disease was more frequently identified in patients with cN1 disease than in patients with cN0 disease (20% [13/64] vs. 7% [24/362]).

### Genomic factors associated with pathologic LN metastasis

**Fig. 1:**
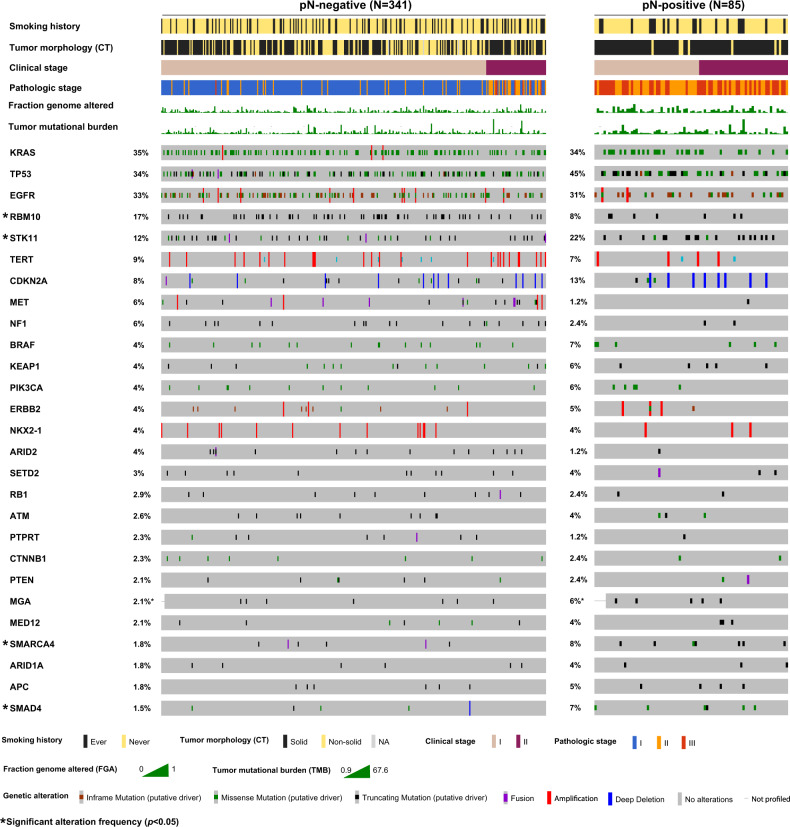
OncoPrint of genes altered in ≥2% of the entire cohort according to pathologic lymph node status. CT computed tomography, NA not available.

Next, we investigated genomic factors associated with pathologic LN metastasis. Among genes altered in ≥2% of the entire cohort (*n* = 27), the most frequently altered gene in pN-negative tumors was *KRAS* (35%)—compared with 34% in pN-positive tumors (Fig. 1). The most frequently altered gene in pN-positive tumors was *TP53* (45%)—compared with 34% in pN-negative tumors. Three genes were altered at significantly higher frequencies in pN-positive than in pN-negative tumors: *STK11* (22% vs. 12%; *p* = 0.024), *SMARCA4* (8% vs. 1.8%; *p* = 0.006), and *SMAD4* (7% vs. 1.5%; *p* = 0.011) (Fig. 1). The alteration frequency of *RBM10* was significantly higher in pN-negative tumors than in pN-positive tumors (17% vs. 8%; *p* = 0.044) (Fig. 1). These gene alterations are mainly driven by the ever-smoker cohort, as there is no statistically significant difference between pN-positive never-smokers and pN-negative never-smokers for *STK11*, *RBM10*, *SMAD4*, and *SMARCA4* (Supplementary Fig. 1). Interestingly, no significant differences in targetable LUAD alterations (*n* = 9) were identified (Supplementary Fig. 2).

**Fig. 2:**
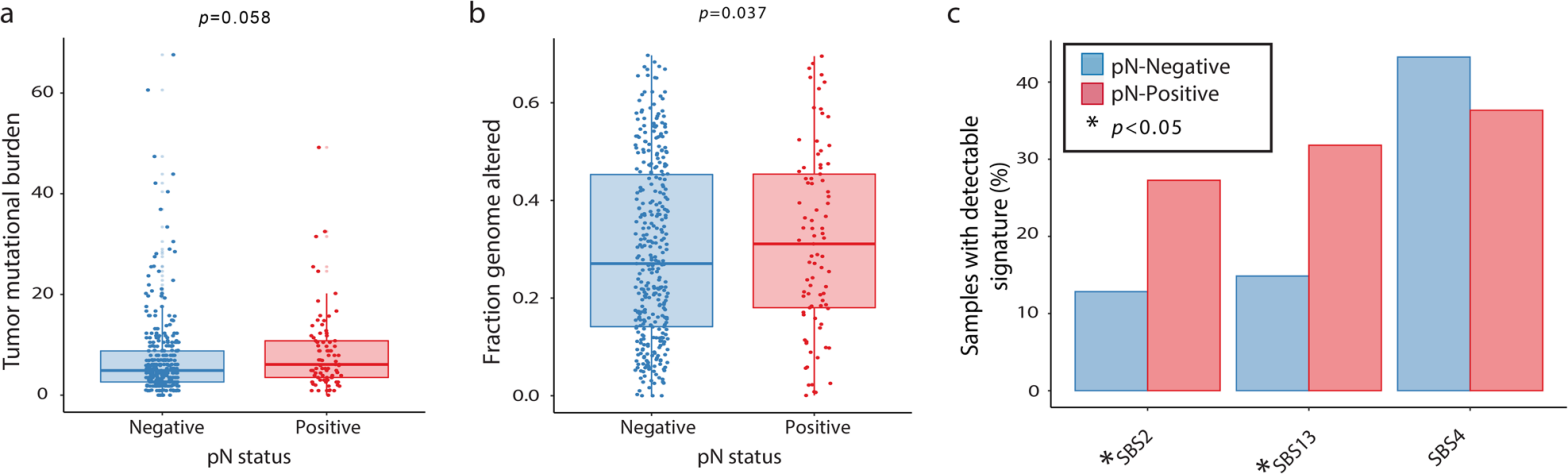
Summary genomic metrics according to pathologic lymph node metastasis. **a** Boxplot of tumor mutational burden (mutations/megabase) versus pathologic lymph node status. **b** Boxplot of fraction of genomic altered versus pathologic lymph node status. **c** Bar graphs showing percent of total tumor samples with most frequently altered mutational signatures (SBS2, SBS13, SBS4) according to pathologic lymph node metastasis (asterisk (*) signifies *p* < 0.05). In the boxplots in this figure, the center line represents the median value, the bounds of the box represent the interquartile range, and the whiskers extend to 1.5× the interquartile range on either side of the median.

The distribution of tumor mutational burden (TMB) did not significantly differ between pN-negative tumors (median [IQR], 4.9 [2.6–8.8] mutations/megabase [Mb]) and pN-positive tumors (median [IQR], 6.1 [3.5–10.8] mutations/Mb; *p* = 0.058) (Fig. 2a). However, fraction of genome altered (FGA) was significantly higher in pN-positive tumors (median [IQR], 0.343 [0.171–0.50] vs. 0.269 [0.141–0.443]; *p* = 0.037) (Fig. 2b).

Additionally, we evaluated the mutational signature profiles in pN-positive and pN-negative tumors. The three most frequent signatures were the smoking signature (SBS4) and the two APOBEC signatures (SBS2 and SBS13). A statistically significantly higher percentage of pN-positive tumors (SBS2 27.3%; SBS13 31.8%) had APOBEC signatures present, compared with pN-negative tumors (SBS2 12.8%; SBS13 14.9%). Both SBS2 (*p* = 0.03) and SBS13 (*p* = 0.02) were statistically significantly more enriched in pN-positive tumors. There was no statistically significant difference in smoking signatures between the two groups (Fig. 2c).

### Copy number alterations associated with pathologic LN metastasis

**Fig. 3:**
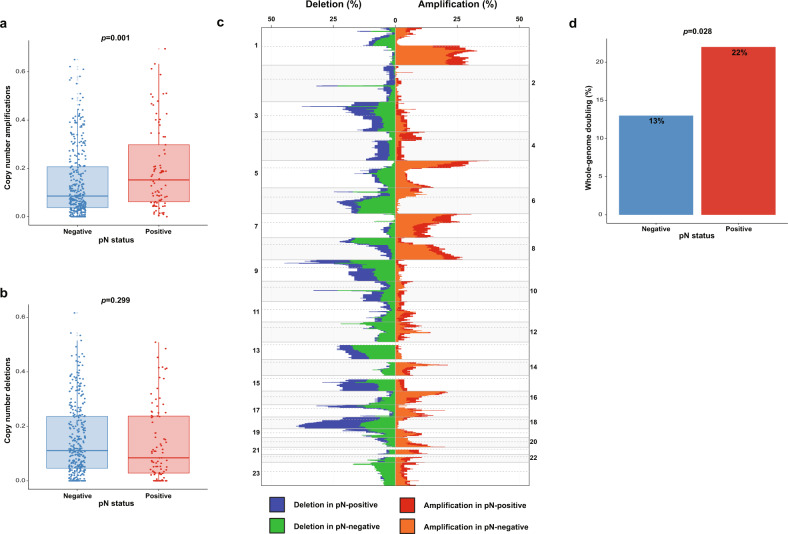
Copy number alteration analysis according to pathologic lymph node metastasis. **a** Boxplot of copy number amplifications versus pathologic lymph node status. **b** Boxplot of copy number deletions versus pathologic lymph node status. **c** Comparison of copy number alterations across chromosome arms according to pathologic lymph node status. **d** Bar plot of the rate of whole-genome doubling versus pathologic lymph node status. In the boxplots in this figure, the center line represents the median value, the bounds of the box represent the interquartile range, and the whiskers extend to 1.5× the interquartile range on either side of the median.

To further examine our observation that pN-positive tumors had significantly higher FGA, we next investigated the copy number landscape (Fig. 3). No significant differences in tumor purity were identified between pN-negative and pN-positive tumors (*p* = 0.059) (Supplementary Fig. 3A). Copy number amplifications were significantly higher in pN-positive tumors than in pN-negative tumors (median [IQR], 0.152 [0.060–0.314] vs. 0.085 [0.037–0.208]; *p* = 0.001) (Fig. 3a). Copy number deletions were not significantly different between groups (*p* = 0.299) (Fig. 3b). Significant differences in chromosome arm-level copy number (false-discovery rate [FDR] *p* < 0.2) between pN-negative and pN-positive tumors were identified at 2p (*p* = 0.122) and 2q (*p* = 0.122), where both arms exhibit more copy number changes in pN-positive tumors (Fig. 3c). The copy number changes observed were broad, and no focal copy number changes were observed on chromosome 2 in either pN-positive or pN-negative tumors.

Genes with copy number alterations present in ≥1% of the entire cohort (*n* = 14 genes; Supplementary Fig. 3B) were also evaluated. Among pN-positive tumors, *CDKN2A* was the most frequently altered (9%), whereas *MDM2* was most frequently altered (10%) among pN-negative tumors. *CDK4* was altered significantly more often in pN-negative tumors than in pN-positive tumors (8% vs. 1.2%; *p* = 0.029) (Supplementary Fig. 3B), and *CDKN2A* was altered significantly more often in pN-positive tumors than in pN-negative tumors (9% vs. 4%; *p* = 0.039) (Supplementary Fig. 3B). Finally, pN-positive tumors had a higher rate of whole-genome doubling (WGD; 22% vs. 13%; *p* = 0.028) (Fig. 3d), further elucidating their chromosomal instability.

### Preoperative clinicopathologic and genomic features associated with pathologic LN metastasis

Next, we performed logistic regression analyses to identify clinicopathologic and genomic features associated with pathologic LN metastasis. Univariable logistic regression analysis identified the following preoperative clinicopathologic variables (Supplementary Table 2): solid versus nonsolid tumor morphologic appearance on CT (*p* < 0.001), tumor size on CT (*p* = 0.001), tumor SUVmax (*p* < 0.001), cN1 versus cN0 (*p* < 0.001), and clinical stage II versus I (*p* < 0.001). We then performed clinical stage-adjusted univariable logistic regression analyses to identify genomic variables associated with pathologic LN metastasis (Supplementary Table 3). The following genomic variables were considered in the final multivariable model: WGD (*p* = 0.014), copy number amplifications (*p* = 0.002), *STK11* alteration versus wild-type (*p* = 0.026), *SMARCA4* alteration versus wild-type (*p* = 0.013), and *SMAD4* alteration versus wild-type (*p* = 0.004).

**Fig. 4:**
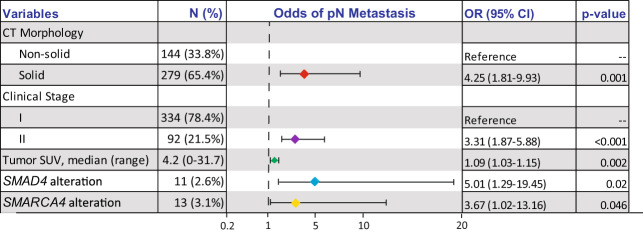
Multivariable logistic regression model of preoperative clinicopathologic and genomic features associated with pathologic lymph node metastasis. In the forest plot in this figure, the points represent the odds ratio, and the whiskers represent the confidence interval corresponding to the table to the right. CI confidence interval, CT computed tomography, OR odds ratio, SUV standardized uptake value.

On multivariable regression analysis combining preoperative clinicopathologic and genomic features, the following variables were independently associated with pathologic LN metastasis (Fig. 4): solid versus nonsolid tumor morphologic appearance on CT (odds ratio [OR], 4.25; 95% confidence interval [CI], 1.81–9.93; *p* = 0.001), tumor SUVmax (OR, 1.09; 95% CI, 1.03–1.15; *p* = 0.002), clinical stage II versus I (OR, 3.31; 95% CI, 1.87–5.88; *p* < 0.001), *SMARCA4* alteration versus wild-type (OR, 3.67; 95% CI, 1.02–13.16; *p* = 0.046), and *SMAD4* alteration versus wild-type (OR, 5.01; 95% CI, 1.29–19.45; *p* = 0.02).

### Association between copy number amplifications and clinicopathologic characteristics

To further examine the association between copy number amplifications and pathologic LN metastasis in the clinical stage-adjusted analysis of genomic variables above, we investigated the relationship between copy number amplification tertiles (low vs. intermediate vs. high) and select clinicopathologic characteristics. Significant associations between copy number amplification tertiles and preoperative clinical characteristics (tumor size on CT, tumor solid morphologic appearance on CT, and primary tumor SUVmax) were identified; copy number amplification tertiles were also shown to be associated with pathologic characteristics obtained from the surgical specimen (pathologic tumor size, micropapillary- or solid-predominant histologic subtype, visceral pleural invasion, lymphovascular invasion, tumor spread through air spaces, pathologic LN metastasis, and pathologic stage) (Supplementary Fig. 4).

## Discussion

The ability to better predict pathologic LN metastasis would allow the identification of subgroups of patients who may benefit from neoadjuvant therapy before tumor resection. In the present study, we found that, of 362 patients with cN0 disease, 15% (*n* = 54) had pathologic LN metastasis at the time of surgery. This results in a false-negative rate (proportion of pN-positive patients who had cN0 disease) of 64% for preoperative clinical LN staging. To address the limitations of radiographic nodal assessment and staging, we analyzed tumor genomic features as well as clinical and radiographic features to determine variables that are independently associated with pathologic LN metastasis in patients with clinical stage I/II LUAD.

Multiple studies have identified radiographic and pathologic predictors of occult LN metastasis, including SUVmax^17^, lymphatic invasion^17^, vascular invasion^2^, and micropapillary histologic pattern^9^. Most of these studies used data that can be reliably obtained only from the pathologic specimen; the utility of such data for preoperative or intraoperative decision-making is therefore limited. Our group previously reported that frozen section analysis can be used to detect micropapillary histologic patterns^18^, the results of which can guide intraoperative decision-making. More recently, we demonstrated that the presence of tumor spread through air spaces on frozen section analysis is an independent predictor of occult pathologic LN metastasis^19^. However, the challenge with the use of pathologic predictors obtained from intraoperative frozen sections or the surgical specimen is that such features are not applicable to preoperative clinical decision-making. Recently, Verdial and colleagues, in a study of patients with clinical stage I-IIIB NSCLC, reported a prediction model that used only radiographic variables and had a bias-corrected C-index of 0.78^15^.

During the last decade, NGS has changed the clinician’s approach to the management of NSCLC^16^. The model developed here combines clinical variables available preoperatively (tumor morphologic appearance on CT, tumor SUVmax, and clinical stage) and genomic data (*SMAD4* and *SMARCA4* alterations) that can be obtained from a preoperative biopsy specimen to better guide the therapeutic strategy. A similar methodology has recently been used to individualize therapy for patients with melanoma^20^.

We evaluated various genomic summary metrics, such as TMB and FGA, as well as individual gene alterations for inclusion in the final pathologic LN metastasis prediction model. Alterations in *SMAD4* and *SMARCA4* were independently associated with pathologic LN metastasis. Both *SMAD4* and *SMARCA4* were altered at significant rates in tumors from patients with pathologic LN metastasis. *SMAD4* mediates signaling of transforming growth factor beta and bone morphogenic protein ligands, and it is a well-defined tumor suppressor in pancreatic and colon cancer^21,22^. Reduced *SMAD4* expression in NSCLC has been associated with increased DNA damage, reduced DNA repair, and increased sensitivity to topoisomerase inhibitors^23,24^. *SMARCA4* is a subunit of the switch/sucrose nonfermentable (SWI/SNF) complex, which plays important roles in chromatin remodeling and, thus, in the regulation of vital cellular processes and functions, such as gene expression, proliferation, and differentiation^25^. In lung cancer, *SMARCA4* inactivation is the most common alteration within the SWI/SNF complex and has been associated with poor oncologic outcomes^26,27^. Recently, Schoenfeld and colleagues reported that *SMARCA4* alterations co-occurred more frequently with *KRAS*, *STK11*, and *KEAP1* mutations, compared with *SMARCA4* wild-type tumors^27^. Interestingly, they also identified improved outcomes after treatment with immunotherapy in patients with *SMARCA4*-mutant tumors^27,28^.

We also investigated the genomic landscape of tumors from patients with pathologic LN metastasis and patients without pathologic LN metastasis. Alteration rates of *STK11*, *SMARCA4*, and *SMAD4* were significantly higher among tumors from patients with pathologic LN metastasis, consistent with prior findings^29^. In our cohort, the increased alteration rates are mainly driven by the ever-smoker subgroup, as these genes are not frequently altered in the never-smokers. This is likely caused by the small sample size of never-smokers, especially pN-positive never-smokers, and the lower TMB burden in the never-smokers (Supplementary Fig. 1). A more comprehensive investigation with larger numbers of never-smoker patients is needed to assess differences between pN-positive ever-smokers and never-smokers.

FGA and copy number amplifications were also statistically significantly higher among tumors from patients with pathologic LN metastasis. FGA is a surrogate for chromosome instability, which has been shown to promote tumor metastasis through the activation of the cGAS-STING pathway^30,31^. FGA has been shown to correlate with survival in other cancers^32–36^. A higher level of subclonal copy number alterations has been associated with poorer disease-free survival in patients with resected early-stage NSCLC^37^.

Additionally, we found an enrichment in APOBEC signatures in pN-positive tumors. The APOBEC signatures have been shown to contribute to increased tumor heterogeneity in both primary^38^ and metastatic NSCLC tumors^39^. These findings have important clinical implications, as APOBEC signatures have the potential to predict immune response, which may serve as a potential marker for immunotherapy in pN-positive patients^40^.

Interestingly, we found an association between copy number amplifications and pathologic LN metastasis on univariable analysis, which prompted us to further investigate the relationship between copy number amplification and clinicopathologic variables. We identified a significant relationship between high copy number amplification tertiles and various poor clinicopathologic indicators. Finally, as further evidence of their more aggressive nature, tumors from patients with pathologic LN metastasis were associated with a higher rate of WGD, which has been associated with poor long-term survival across multiple cancers^41^.

This study has several limitations. NGS was performed using single-region sampling of the primary tumor. As previously noted, intratumoral heterogeneity is intrinsic to LUAD^37^, and single-region sampling may not accurately capture the complexity of the disease, such as its clonal architecture^42^. In addition, tumor genomic analyses were performed on the surgical specimen, not on preoperative biopsy specimens. Obtaining high-quality DNA for NGS from biopsy specimens can be a challenge^43^, but success rates of 80–90% are now being reported for small tumor tissue samples obtained from CT-guided and bronchoscopic biopsies—convincingly demonstrating that acquisition of adequate quality DNA for NGS is possible before surgical resection^44^. Our final model incorporates *SMARCA4* and *SMAD4* alterations; however, similar to many other genomic drivers of aggressive tumor biology, the frequency of these alterations is modest, especially in never-smokers^45^. Finally, external validation is required to evaluate the performance of the model.

In summary, this study highlights the potential importance of genomic data for identification of patients at risk of pathologic LN metastasis. Although clinical stage remains important for identifying patients at risk of pathologic LN metastasis, in isolation it does not perform well in the prediction of nodal disease in this early-stage LUAD cohort. Our final multivariable model comprised preoperative clinical features and *SMARCA4* and *SMAD4* alteration data that were found to be independently associated with pathologic LN metastasis. The ability to identify patients with stage I/III LUAD who are at high risk of pathologic LN metastasis could potentially guide the therapeutic strategy prior to surgical resection.

## Methods

### Patient cohort

This study was approved by the institutional review board at Memorial Sloan Kettering Cancer Center. All patients provided written informed consent to participate in the institutional review board–approved protocol. Patients included in the study underwent complete resection for LUAD and had NGS (Memorial Sloan Kettering–Integrated Mutation Profiling of Actionable Cancer Targets [MSK-IMPACT]^46^) performed on their primary tumor between 2010 and 2018. All patients received an anatomic resection (lobectomy, segmentectomy, or pneumonectomy) with LN dissection. Exclusion criteria included induction therapy, wedge resection, microscopic or macroscopic residual disease (R1/R2 resection), and low-quality NGS. Patients included in the study were grouped according to pathologic LN metastasis (see CONSORT diagram, Supplementary Fig. 5). pN1 and pN2 patients were grouped together as there were no statistically significant differences in gene frequency alterations between pN1 and pN2 patients.

Clinical characteristics, preoperative CT and PET, and pathology reports (adjusted according to the 8th edition of the AJCC Cancer Staging Manual) were reviewed. Tumor size, presence of solid tumor morphologic appearance, primary tumor SUVmax, and lymphadenopathy (documented as such or ≥1 cm in short axis on CT scan^15^) were recorded. Follow-up was performed in accordance with National Comprehensive Cancer Network guidelines^47^.

### Tumor genomic analysis

MSK-IMPACT sequencing was performed and analyzed as previously described^46,48^. TMB was defined as the fraction of nonsynonymous single-nucleotide or insertion/deletion mutations divided by the length of the coding region (in Mb) sequenced by each panel (0.98, 1.06, and 1.22 Mb in the 341-, 410-, and 468-gene panels, respectively). FGA was computed from the output of Fraction and Allele-Specific Copy Number Estimates from Tumor Sequencing (FACETS), which provides accurate, purity- and ploidy-corrected, integer DNA copy number calls from sequenced samples. FGA is defined as the fraction of the genome that differs from the major integer copy number (MCN), which is defined as the integer total copy number spanning the largest portion of the genome^49^.

Copy number alteration frequency plots were generated using the Integrative Genomics Viewer from Broad Institute. Significant focal copy number alterations were identified from segmented data using GISTIC 2.0^50^. Copy number deletions, amplifications, WGD, and arm-level FGA estimates were calculated from the FACETS method output^49^. Copy number amplifications were defined as the fraction of the genome that was greater than the MCN, whereas copy number deletions were defined as the fraction of the genome that was less than the MCN. Tumor samples were considered to have undergone WGD if >50% of their autosomal genome had an MCN (the more frequent allele in a given segment) >2. Arm-level FGA was defined as the fraction of the chromosomal arm that differed from the MCN. *p*-values highlighting differences in arm-level FGA between histologic subtypes were calculated using the Kruskal–Wallis test and were adjusted for multiple comparisons using the FDR method; FDR *p* < 0.2 was considered significant.

Mutational signatures were computed^51^ for samples with at least 13.8 Mut/Mb, as described by Zehir et al.^52^ (pN-positive [*n* = 44] and pN-negative [*n* = 141]). We used the most recent version of the single base substitution signatures defined in the Catalogue of Somatic Mutations in Cancer database^53^. Samples were considered to have a signature present if the mean signature value was greater than 0.1.

### Statistical analysis

The Chi-square test and Mann–Whitney U-test were used to compare the categorical and continuous factors between the two patient cohorts (pN-negative [pN0] vs. pN-positive [pN1/pN2]), respectively. Fisher’s exact test was used to compare the alteration frequencies of genes altered in ≥2% of the entire cohort. Univariable logistic regression analysis was performed to quantify the relationships between preoperative clinicopathologic features and pathologic LN metastasis. A separate univariable logistic regression analysis, adjusted for clinical stage, was performed to quantify the relationships between genomic features and pathologic LN metastasis. A multivariable logistic regression model was constructed starting with preoperative clinicopathologic and genomic factors with *p* < 0.1 in univariable analyses in a backward-selection method. All analyses were two-sided, and *p* < 0.05 was considered to indicate statistical significance. All analyses were performed using Stata 15.0 (StataCorp, College Station, TX) and R 3.5.3 (R Core Team, Vienna, Austria).

### Reporting summary

Further information on research design is available in the Nature Research Reporting Summary linked to this article.

## Supplementary information

Supplementary Information

Reporting Summary

## Data Availability

All the genomic and clinical data used in our analyses are publicly available through the cBioPortal for Cancer Genomics^54^ at https://www.cbioportal.org/study/summary?id=luad_msk_npjpo_2021.
