# Supplementary material for: Preoperative clinical and tumor genomic features associated with pathologic lymph node metastasis in clinical stage I and II lung adenocarcinoma
Source: NPJ Precis Oncol. 2021 Jul 21;5:70. doi: 10.1038/s41698-021-00210-2 (PMC8295366; doi:10.1038/s41698-021-00210-2)
Supplement: Supplementary file 1 — Supplementary Information [file 41698_2021_210_MOESM1_ESM.pdf]

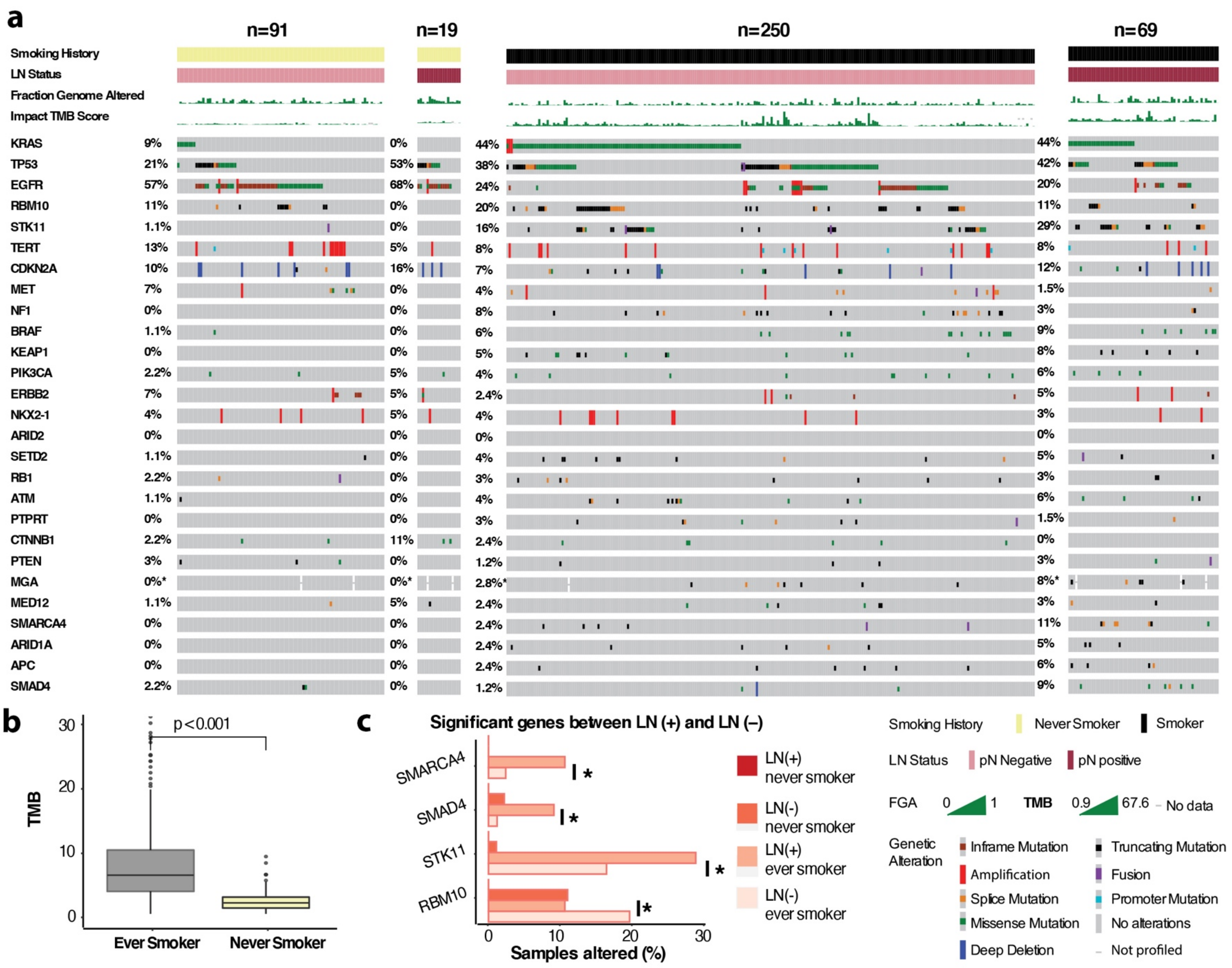
a
n=91
n=19
n=250
n=69
Smoking History
LN Status
Fraction Genome Altered
Impact TMB Score
KRAS 9% 0% 44% 44%
TP53 21% 53% 38% 42%
EGFR 57% 68% 24% 20%
RBM10 11% 0% 20% 11%
STK11 1.1% 0% 16% 29%
TERT 13% 5% 8% 8%
CDKN2A 10% 16% 7% 12%
MET 7% 0% 4% 1.5%
NF1 0% 0% 8% 3%
BRAF 1.1% 0% 6% 9%
KEAP1 0% 0% 5% 8%
PIK3CA 2.2% 5% 4% 6%
ERBB2 7% 5% 2.4% 5%
NKX2-1 4% 5% 4% 3%
ARID2 0% 0% 0% 0%
SETD2 1.1% 0% 4% 5%
RB1 2.2% 0% 3% 3%
ATM 1.1% 0% 4% 6%
PTPRT 0% 0% 3% 1.5%
CTNNB1 2.2% 11% 2.4% 0%
PTEN 3% 0% 1.2% 3%
MGA 0%* 0%* 2.8%* 8%*
MED12 1.1% 5% 2.4% 3%
SMARCA4 0% 0% 2.4% 11%
ARID1A 0% 0% 2.4% 5%
APC 0% 0% 2.4% 6%
SMAD4 2.2% 0% 1.2% 9%
b
TMB
p<0.001
30
20
10
0
Ever Smoker
Never Smoker
c
Significant genes between LN (+) and LN (–)
SMARCA4
SMAD4
STK11
RBM10
*
0
10
20
30
Samples altered (%)
LN(+) never smoker
LN(-) never smoker
LN(+) ever smoker
LN(-) ever smoker
Smoking History
Never Smoker
Smoker
LN Status
pN Negative
pN positive
FGA 0 1
TMB 0.9 67.6
No data
Genetic Alteration
Inframe Mutation
Amplification
Splice Mutation
Missense Mutation
Deep Deletion
Truncating Mutation
Fusion
Promoter Mutation
No alterations
Not profiled

Supplementary Figure 1. **a** OncoPrint of fraction genome altered, tumor mutation burden, and the most commonly altered genes (≥2% of the overall cohort) broken down by smoking history and lymph node status. **b** Comparison of tumor purity according to smoking history. In the box plots in this figure, the center line represents the median value, the bounds of the box represent the interquartile range, and the whiskers extend to 1.5 × the interquartile range on either side of the median. **c** Genes significantly associated with survival after univariable analysis stratified by smoking history and lymph node status.

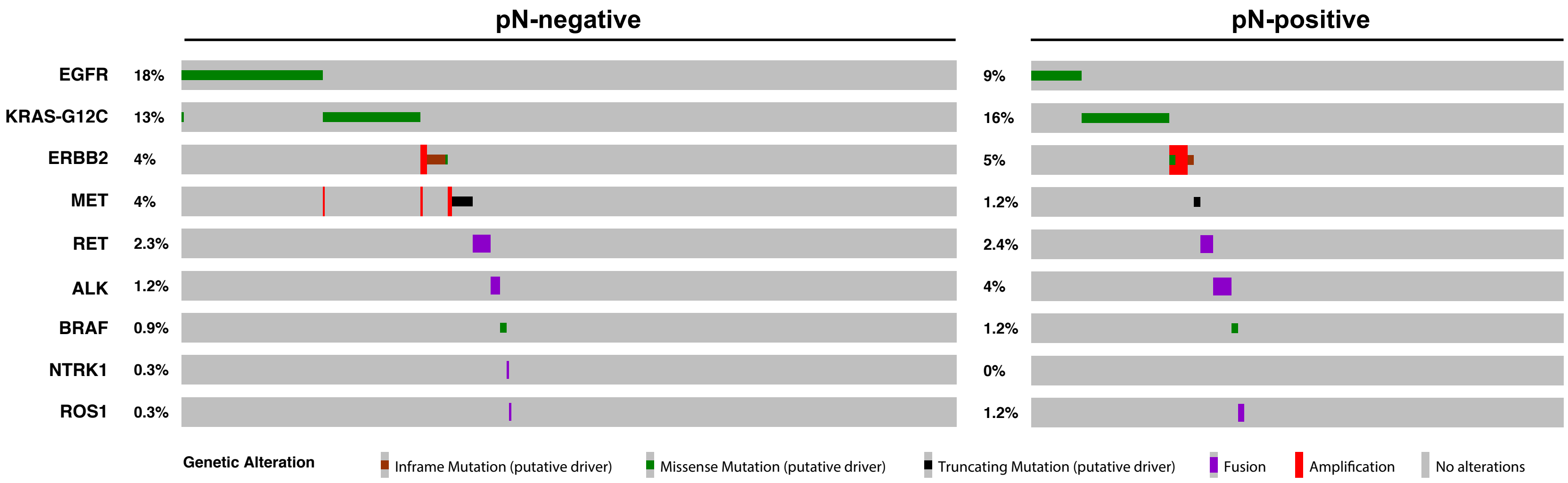

Supplementary Figure 2. OncoPrint of targetable lung adenocarcinoma genes according to pathologic lymph node metastasis.

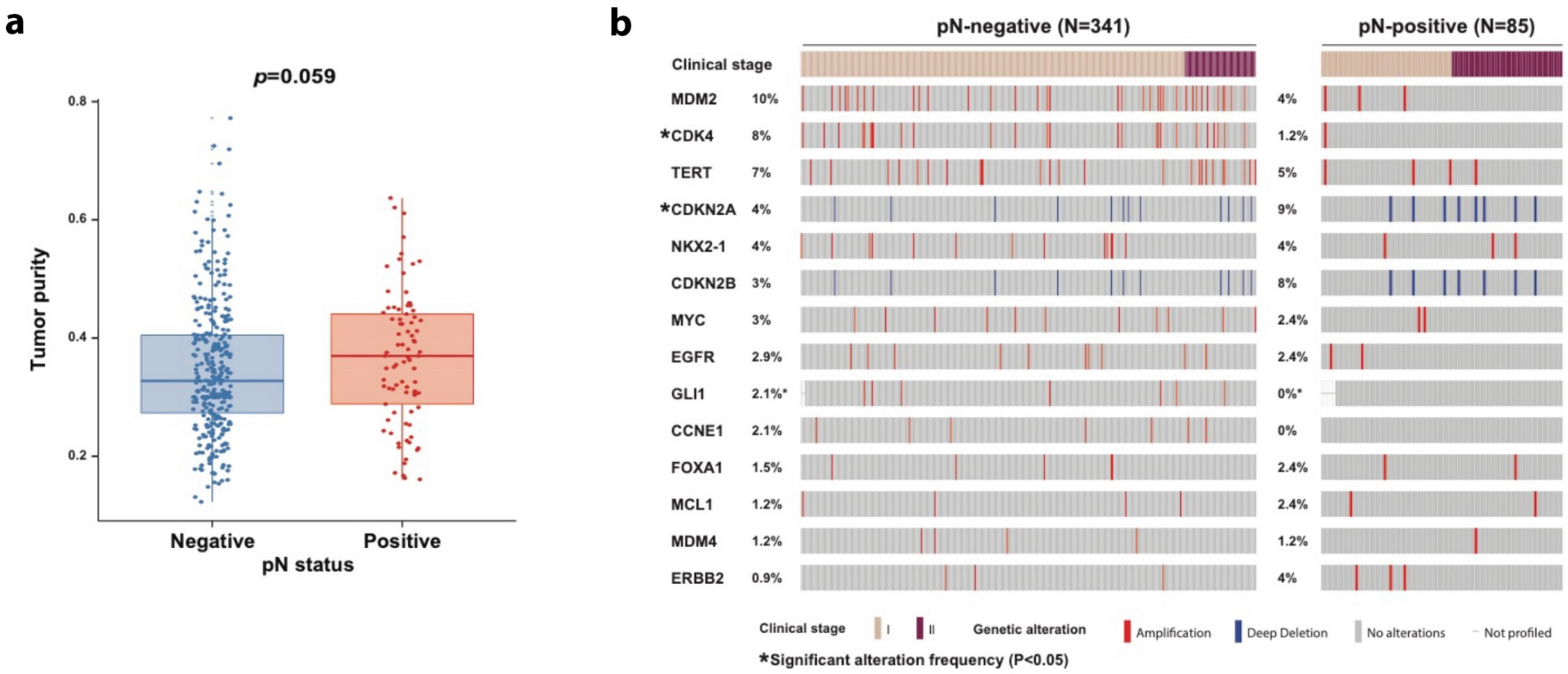

Supplementary Figure 3. Comparison of tumor purity (**a**) and OncoPrint of copy number genes altered in ≥1% of the entire cohort (**b**) according to pathologic lymph node metastasis. In the box plots in this figure, the center line represents the median value, the bounds of the box represent the interquartile range, and the whiskers extend to 1.5 × the interquartile range on either side of the median.

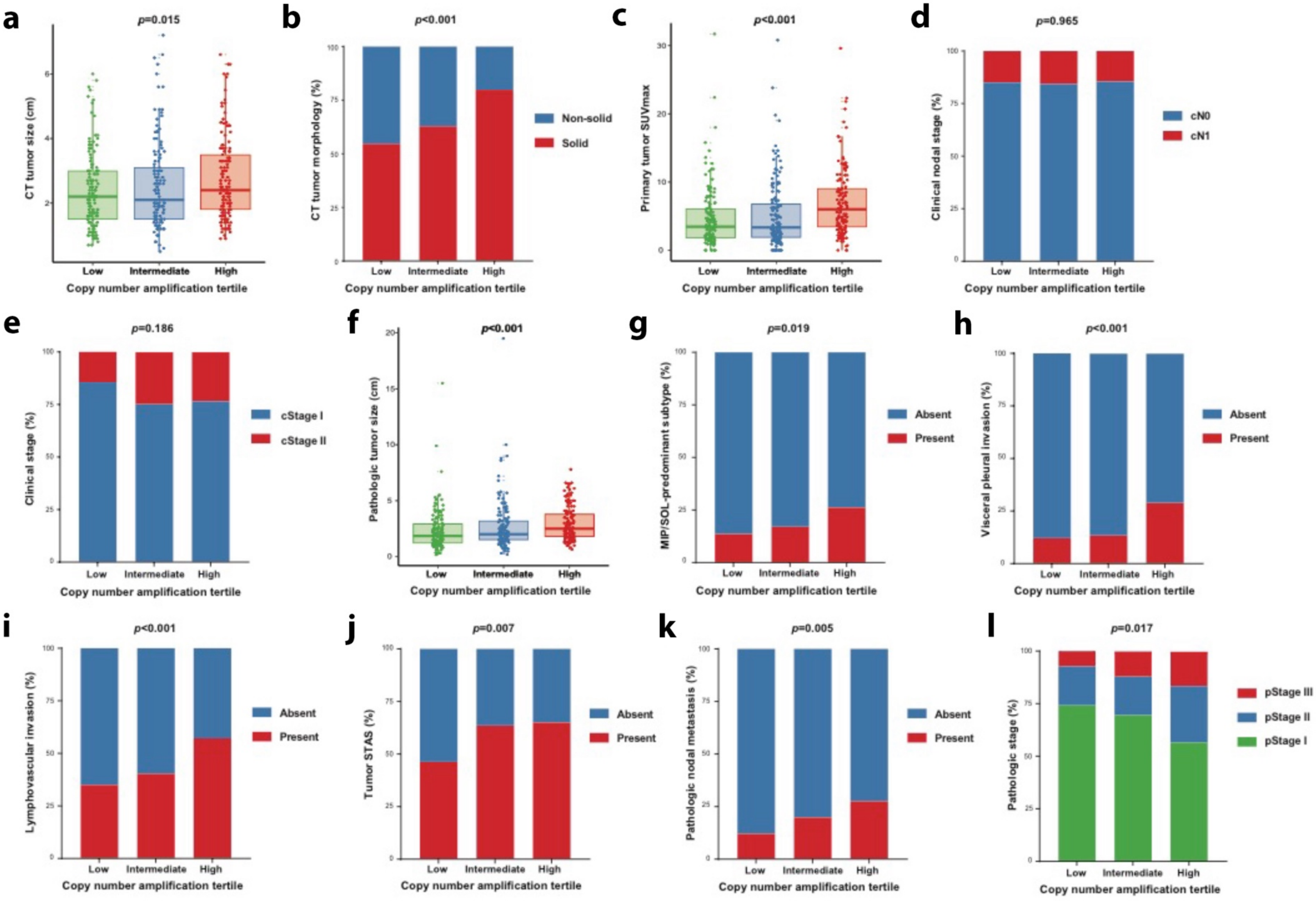
a
p=0.015
CT tumor size (cm)
Low
Intermediate
High
Copy number amplification tertile
b
p<0.001
CT tumor morphology (%)
Non-solid
Solid
c
p<0.001
Primary tumor SUVmax
d
p=0.965
Clinical nodal stage (%)
cN0
cN1
e
p=0.186
Clinical stage (%)
cStage I
cStage II
f
p<0.001
Pathologic tumor size (cm)
g
p=0.019
MIP/SOL-predominant subtype (%)
Absent
Present
h
p<0.001
Visceral pleural invasion (%)
i
p<0.001
Lymphovascular invasion (%)
j
p=0.007
Tumor STAS (%)
k
p=0.005
Pathologic nodal metastasis (%)
l
p=0.017
Pathologic stage (%)
pStage III
pStage II
pStage I

Supplementary Figure 4. Association of copy number amplification tertiles and clinicopathologic variables. **a** Box plot of copy number amplification tertiles versus tumor size on computed tomography (CT). **b** Stacked bar plot of copy number amplification tertiles versus CT tumor morphologic appearance. **c** Box plot of copy number amplification tertiles versus primary tumor maximum standardized uptake value (SUVmax). **d** Stacked bar plot of copy number amplification tertiles versus clinical lymph node (cN) status. **e** Stacked bar plot of copy number amplification tertiles versus clinical stage. **f** Box plot of copy number amplification tertiles versus pathologic tumor size. **g** Stacked bar plot of copy number amplification tertiles versus micropapillary (MIP)– or solid (SOL)–predominant histologic subtype in pathologic specimen. **h** Stacked bar plot of copy number amplification tertiles versus visceral pleural invasion. **i** Stacked bar plot of copy number amplification tertiles versus lymphovascular invasion. **j** Stacked bar plot of copy number amplification tertiles versus tumor spread through air spaces (STAS). **k** Stacked bar plot of copy number amplification tertiles versus pathologic nodal metastasis. **l** Stacked bar plot of copy number amplification tertiles versus pathologic stage. In the box plots in this figure, the center line represents the median value, the bounds of the box represent the interquartile range, and the whiskers extend to 1.5 × the interquartile range on either side of the median.

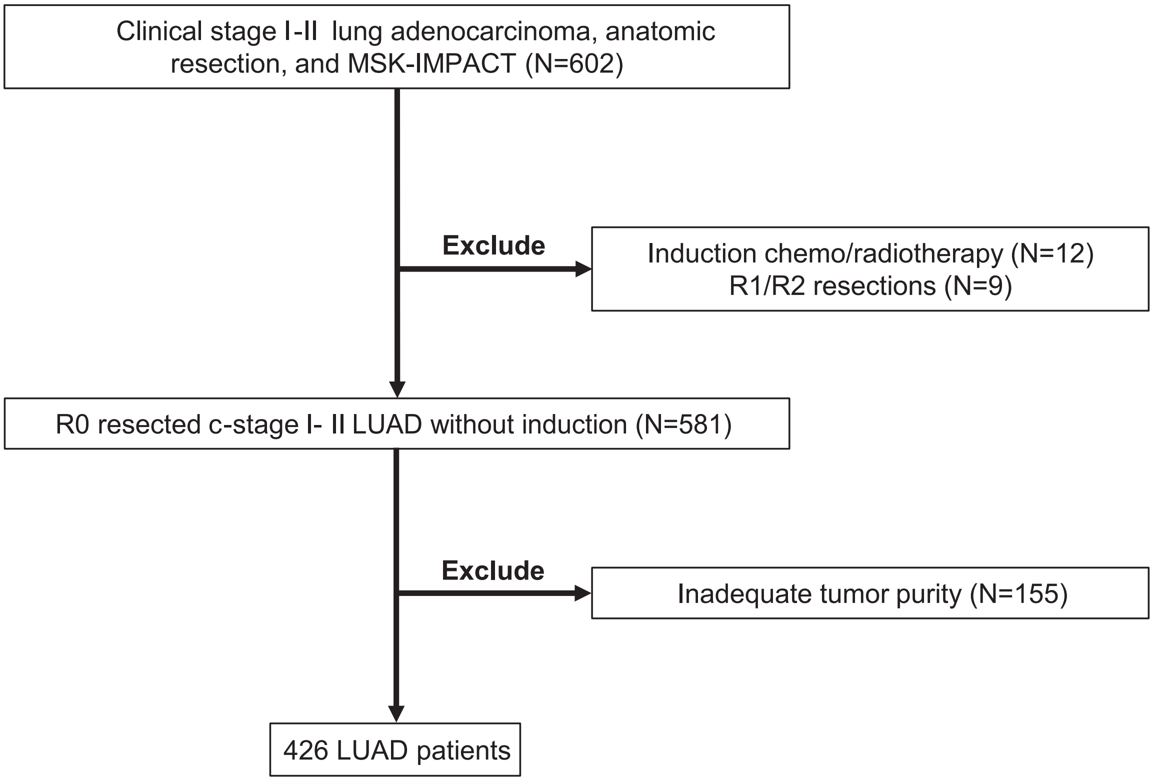

Supplementary Figure 5. CONSORT diagram. LUAD, lung adenocarcinoma.

Supplementary Table 1. Total lymph nodes and lymph node stations sampled according to pathologic lymph node metastasis

LNs	Total (N=426)	pN-negative (N=341)	pN-positive (N=85)
Total LNs sampled	15 (11-21)	14 (11-20)	17 (11.5-26)
N1 stations sampled	3 (2-3)	3 (2-3)	3 (2-3)
N2 stations sampled	3 (2-3)	3 (2-3)	3 (2-3)
Total LN stations sampled	5 (4-6)	5 (4-6)	5 (4-6)

Data are presented as median (interquartile range).

Abbreviation: LN, lymph node.

Supplementary Table 2. Univariable logistic regression analysis for the association of preoperative clinicopathologic variables with pathologic lymph node metastasis

Variable	OR	95% CI	*p* value
Age at resection	1.00	0.97-1.03	0.9
Male sex (vs. female)	0.62	0.39-1.00	0.052
Ever smoking status (vs. never)	0.79	0.45-1.39	0.4
Smoking pack-years	1.00	0.99-1.01	0.5
Solid tumor morphologic appearance on CT (vs. nonsolid)	7.59	3.40-16.96	<0.001
Tumor size on CT	1.35	1.14-1.61	0.001
Tumor SUVmax	1.15	1.09-1.20	<0.001
cN1 (vs. cN0)	5.36	3.03-9.47	<0.001
Clinical stage II (vs. I)	4.61	2.75-7.73	<0.001
Biopsy contains MIP	1.05	0.42-2.62	0.9
Biopsy contains SOL	1.74	0.66-4.56	0.3

Abbreviations: CI, confidence interval; CT, computed tomography; MIP, micropapillary; OR, odds ratio; SOL, solid; SUV, standardized uptake value.

Supplementary Table 3. Clinical stage–adjusted univariable logistic regression analysis for the association of genomic variables with pathologic lymph node metastasis

Variable	OR	95% CI	*p*
TMB	1.01	0.98-1.04	0.6
FGA	1.67	0.43-6.47	0.5
WGD	2.21	1.17-4.18	0.014
Copy number deletions	0.57	0.08-3.82	0.6
Copy number amplifications	11.20	2.38-52.7	0.002
TP53	1.54	0.93-2.55	0.1
KRAS	0.85	0.50-1.44	0.5
EGFR	1.03	0.60-1.77	0.9
RBM10	0.49	0.21-1.14	0.10
STK11	2.07	1.09-3.91	0.026
TERT	0.64	0.25-1.66	0.4
CDKN2A	1.81	0.82-3.99	0.14
NF1	0.45	0.10-2.04	0.3
BRAF	1.52	0.54-4.27	0.4
KEAP1	1.60	0.54-4.76	0.4
MET	0.20	0.03-1.61	0.13
PIK3CA	1.40	0.45-4.37	0.6
ERBB2	1.48	0.44-4.97	0.5
NKX2-1	0.76	0.20-2.90	0.7
ARID2	0.20	0.02-1.62	0.13
SETD2	1.11	0.28-4.34	0.9
SMARCA4	4.49	1.37-14.74	0.013
MGA	2.36	0.67-8.27	0.2
ATM	1.11	0.27-4.54	0.9
RB1	0.73	0.15-3.63	0.7
SMAD4	6.40	1.81-22.62	0.004
MED12	1.58	0.37-6.79	0.5
APC	2.22	0.56-8.81	0.3
CTNNB1	0.85	0.16-4.43	0.8
PTPRT	0.46	0.05-4.01	0.5
ARID1A	1.78	0.40-7.94	0.5
PTEN	1.15	0.22-6.10	0.9

Gene names are in italic type.

Abbreviations: CI, confidence interval; FGA, fraction of genome altered; OR, odds ratio; TMB, tumor mutational burden; WGD, whole-genome doubling.
